# Correction of copy number induced false positives in CRISPR screens

**DOI:** 10.1371/journal.pcbi.1006279

**Published:** 2018-07-19

**Authors:** Antoine de Weck, Javad Golji, Michael D. Jones, Joshua M. Korn, Eric Billy, E. Robert McDonald, Tobias Schmelzle, Hans Bitter, Audrey Kauffmann

**Affiliations:** 1 Novartis Institutes for Biomedical Research, Basel, Switzerland; 2 Novartis Institutes for Biomedical Research, Cambridge, MA, United States of America; Princeton University, UNITED STATES

## Abstract

Cell autonomous cancer dependencies are now routinely identified using CRISPR loss-of-function viability screens. However, a bias exists that makes it difficult to assess the true essentiality of genes located in amplicons, since the entire amplified region can exhibit lethal scores. These false-positive hits can either be discarded from further analysis, which in cancer models can represent a significant number of hits, or methods can be developed to rescue the true-positives within amplified regions. We propose two methods to rescue true positive hits in amplified regions by correcting for this copy number artefact. The Local Drop Out (LDO) method uses the relative lethality scores within genomic regions to assess true essentiality and does not require additional orthogonal data (e.g. copy number value). LDO is meant to be used in screens covering a dense region of the genome (e.g. a whole chromosome or the whole genome). The General Additive Model (GAM) method models the screening data as a function of the known copy number values and removes the systematic effect from the measured lethality. GAM does not require the same density as LDO, but does require prior knowledge of the copy number values. Both methods have been developed with single sample experiments in mind so that the correction can be applied even in smaller screens. Here we demonstrate the efficacy of both methods at removing the copy number effect and rescuing hits from some of the amplified regions. We estimate a 70–80% decrease of false positive hits with either method in regions of high copy number compared to no correction.

## Author summary

Cancer vulnerabilities have been identified by systematically disrupting individual genes in cancer cells and observing the resulting effect on cell proliferation. In recent years, a new gene editing technique called CRISPR has made it easier and cheaper to disrupt genes by precisely and completely suppressing the function of individual genes by cutting through its DNA. However, an artefact of the approach yields false positives: using CRISPR to target genes in regions of the genome which are abnormally repeated, called copy number alterations (CNA), has been shown to kill the investigated cells irrespective of the true essentiality of the amplified genes. This artefact is a particular issue when studying tumours, since CNAs are common in cancer. Additionally cancer-specific genes are known to selectively drive amplification, making the ability to assess the essentiality of genes in these regions even more important. Here we describe and provide the code to computationally correct for this artefact and recover the true essentiality of CNA genes.

## Introduction

CRISPR based loss-of-function screens have emerged as a powerful tool to interrogate multiple species and models [1]. The technology has been quickly adopted to identify essential genes in cancer, including several cancer cell line screens [2–4]. However, as reported in two studies [5,6] and further discussed by others [7], genes in regions of copy number amplification display strong lethal phenotypes by CRISPR-Cas9 cutting (as opposed to CRISPRi [8]), regardless of the true biological essentiality of the targeted gene. This results in a significant number of false positive hits in samples with large copy number alterations as is often the case in cancer models.

One way of mitigating this problem of false positives would be to simply discard any hits found in amplified regions. This is a viable strategy when considering aggregate profiles [9], but runs the risk of yielding many false negatives when looking at individual hits. Especially when copy number events are an important oncogenic driver and identifying the essential gene in the amplicon is of interest to target discovery [10]. Therefore, to fully leverage CRISPR based screens, it is important to understand and correct for the observed copy number bias. Here, we propose methods to correct for the copy number artefact, while rescuing the true positives within the amplicons. The corresponding R scripts are also provided (https://doi.org/10.6084/m9.figshare.5140057.v3). To the best of our knowledge two other methods have recently been proposed in [11,12].

In this study wee used the data published by Munoz et al. [5], where the copy number artefact has been observed (Fig 1A), i.e. a negative correlation of sensitivity (calculated as Log FC) with copy number. To illustrate the methods, we focused on the astrocytoma cell line SF268 and the gastric cancer cell line MKN45, as these two cell lines have amplicons where the driver has been well characterized, *YAP1* and *MET*, respectively [13–16]. The sgRNA library used targeted 2722 human genes with an average coverage of 20 reagents per gene. In addition, a second screen performed on MKN45, using a different library of genes with a coverage of 10 reagents per gene, was used to evaluate the methods described herein. We then evaluated our methods on the Avana dataset [11].

**Fig 1:**
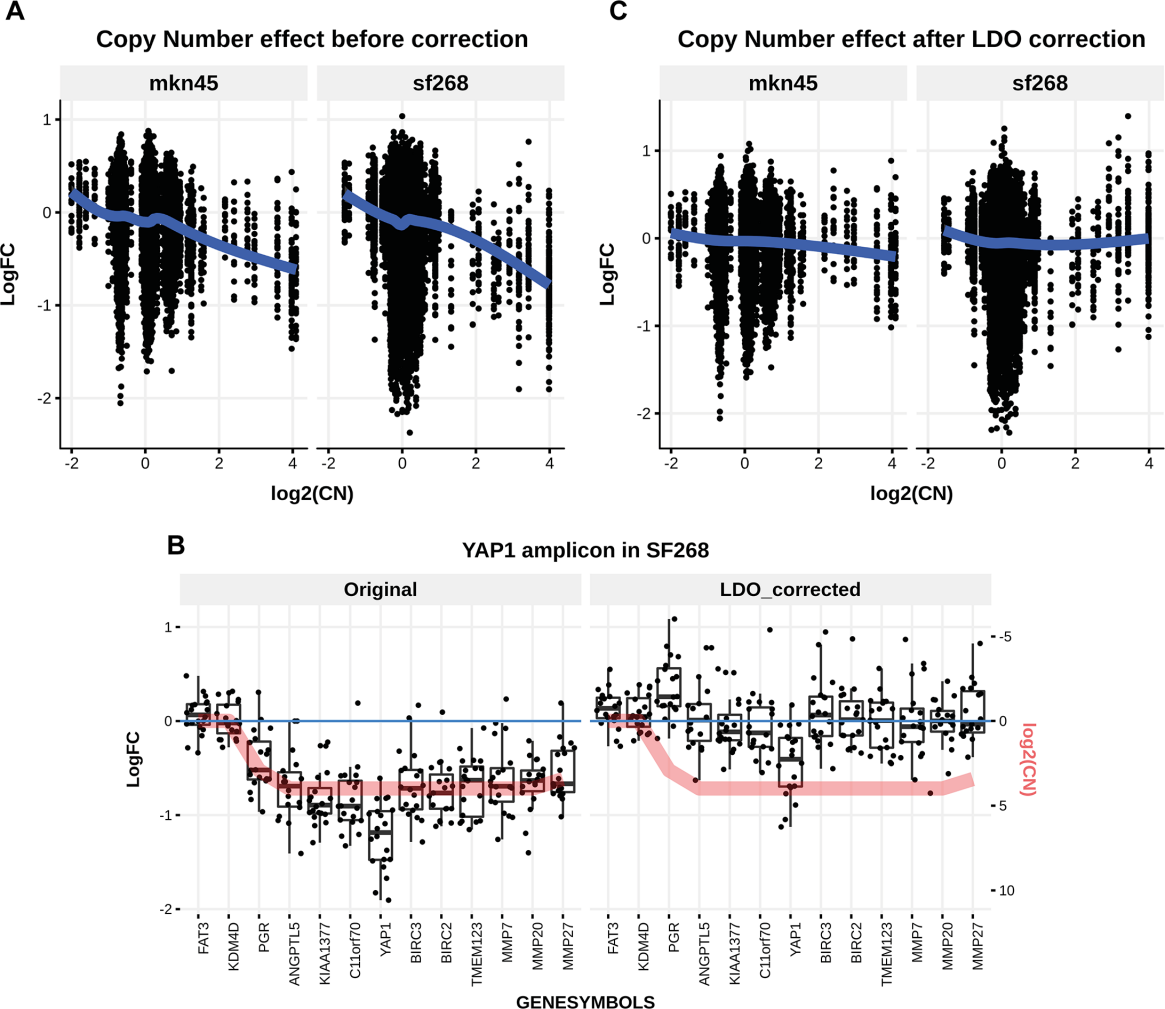
CN effect on CRISPR knock-out sensitivity and LDO correction. **A)** The sensitivity to CRISPR-mediated knock-out is dependent on the level of amplification of the underlying genomic region. Above for MKN45, 84 out of 191 guides in amplified regions (CN of at least 4 (log2(CN) = 2)) score below -0.5, while 274 out of 397 guides score below -0.5 in SF268. **B)** Sensitivity, calculated as LogFC, conferred by each guide (black dots) within the YAP1 amplicon in the SF268 cell line summarized by a boxplot for each gene in the amplicon. To ease the interpretation, the red line displays the inverted copy number value scaled to the data. The left panel a) displays the uncorrected sensitivity scores, while the right panel b) shows the sensitivity scores after LDO correction. **C)** The sensitivity to CRISPR-mediated knock-out after LDO correction is not dependent on the level of DNA amplification of the underlying genomic region anymore.

## Results

### Local Drop Out (LDO) method

To account for the copy number artefact, we propose the Local Drop Out (LDO) method. LDO aims to correct phenotype scores for each guide by taking into account guide scores targeting the other genes in its direct genomic neighbourhood. It assumes that most genes display little or no phenotype upon knock-out in such screens (~2 weeks or less) and does not rely on copy number measurements. If multiple neighbouring genes show similarly strong drop out values by exhibiting a significant reduction of viability score, it is assumed that the observed phenotype is due to a copy number effect rather than a true dependence of the cell line. This assumption is corroborated by observations made in large RNAi screens [17,18] where only a single or few genes are identified as drivers of focal copy number events. The density of the screen influences the size of the copy number events that can be detected: the higher the density of the genes selected to be included in the screen, the more focal the detected copy number events can be.

The LDO method uses a two-step process: 1. A list of potential hits is defined; 2. The remaining “neutral” genes are used to estimate the copy number effect on viability and the viability are corrected based on the estimate. In step one, a list of potential gene hits is defined that minimizes false negatives, and in step two, allows the estimation of copy number effect on viability to be based on “neutral” genes. The potential hit list can be defined in several ways. Prior knowledge can be used, e.g. lists of pan-lethal genes available in the public domain, to determine an initial list of essential guides for consideration. Alternatively, we propose to identify cell line specific genes that are either essential or growth enhancing by calculating each guide’s vulnerability score compared to a weighted mean sensitivity of neighbouring guides not in that gene, i.e. assessing the difference between the dependence score of one guide against the average vulnerability observed in the guides targeting different genes on the same locus. The weighted mean sensitivity is calculated as follows. Let *g* be a guide in the set *G* of all guides in a specific chromosome or chromosomal arm, with $g_{i}^{h}$ the *i*th guide targeting gene *h* and *G*^*h*^ be the set of guides targeting gene *h*. Let *E*_1_ be the set of guides targeting known essential genes. An exponential distribution with parameter *ω* = 100’000 bp is used. Additionally, let the genomic position of guide *g* be *x*_*g*_ and the viability score induced by guide *g* be *S*_*g*_, then the weighted mean sensitivity, excluding essential guides and guides targeting the same gene, $mS(g_{i}^{h})$ for guide $g_{i}^{h}$ can be written as:

$$mS(g_{i}^{h})=\frac{1}{N}\sum_{g\in {G\backslash \{G}^{h}\cup E_{1}\}}S_{g}e^{-\omega |x_{g_{i}^{h}}-x_{g}|}$$

With

$$N=\sum_{g\in {G\backslash \{G}^{h}\cup E_{1}\}}e^{-\omega |x_{g_{i}^{h}}-x_{g}|}$$

For each guide, the first iteration of the corrected sensitivity *S*^1^ value is obtained from subtracting the weighted mean sensitivity for that guide to the original sensitivity value without correction ($S_{g_{i}^{h}}^{1}=S_{g_{i}^{h}}-mS(g_{i}^{h})$). Using this measure, the guides with absolute values above the *μ*^*th*^ percentile across the entire genome are considered as guides displaying potential true phenotypes (hits) and are not used in the second iteration of the method (by default *μ* = 85).

In this analysis, we have used a list of a priori essential genes compiled from [4] (see Mat & Met for more details). Removing the known pan-essential genes is not a requirement of the method, but can improve the accuracy of the resulting CN correction. In particular in the case of successively located pan-essential genes which could otherwise be confused for a CN event. We have used *μ* = 85 since it represents a prior belief that we can expect about 15% of genes (and therefore 15% of guides in our design) to display a true phenotype in the screen independent of the copy number. The parameter can be modified, e.g. one might expect a larger percentage of genes displaying a phenotype in longer screens. This procedure is equivalent to increasing the set of essential guides in set *E*_2_ which is then sample specific and contains the set *E*_1_ and all the guides identified above the *μ*^*th*^ percentile.

In the second step of the LDO correction, all guides below the *μ*^*th*^ percentile are used to fit a regression tree to estimate the copy number effect. These guides are highly enriched in guides showing no phenotypes or “neutral” gene guides, i.e. the set of guides *g* ∈ *G*\*E*_2_. Here, a one dimensional regression tree is used to estimate the sensitivity of the guides as a function of the genomic location alone. The copy number effect identified by the regression is then removed from the original sensitivity score *S*_*g*_ to obtain the LDO corrected sensitivity score $S_{g}^{LDO}$.

Specifically, the regression tree *T* formulates the copy number induced sensitivity *S*_*CN*_ at position *x* as follows:

$${S_{CN}(x|T)=S}_{CN}(x|\{S_{m},R_{m}{\}}_{1}^{M})=\sum_{m=1}^{M}S_{m}1(x\in R_{m})$$

Where $\{R_{m}{\}}_{1}^{M}$ are subregions of the genome, and *x* is a genomic position. *S*_*m*_ are the estimated copy number induced sensitivity values in region *R*_*m*_. Using only the guides g ∈ *G*\*E*_2_, we try to find the regression tree *T* which minimizes the error:

$$e(T)=\sum_{g\in G\backslash E_{2}}{\left[S_{g}-S_{CN}^{T}(x_{g}|S_{m},R_{m})\right]}^{2}$$

with respect to *S*_*m*_ and *R*_*m*_. In practice, a regularization term is added to avoid overfitting. Thus, the objective is to identify the tree *T* which minimizes the following term:

$$\underset{T\in T(\beta )}{\min}\left[e(T)+\alpha |T|\right]$$

where |*T*| is the number of terminal nodes of the tree and the complexity parameter *α* measures the “cost” of adding another region *R*_*m*_ to the model. The higher the cost, the shallower the tree. Also additional constraints can be set on the universe T(β) of potential trees *T*. In particular, one can consider the universe T(β) with a minimum number *β* of guides per region *R*_*m*_.

The regression is performed iteratively with increasing values of the cost parameter *α* and constant value of *β*. By default the parameter *α* is initialised to 10^−*k*^ with *k* = 3 and *β* is set to twice the mean number of guides per gene. The value *k* is iteratively decreased in increments *i*_*k*_ set by default to 0.1. This process decreases the complexity of the regression tree until all regions $R^{F}\in \{R_{m}{\}}_{1}^{M}$ contain at least 3 genes. The last resulting tree *T*^*LDO*^ is used to calculate the LDO corrected sensitivity scores $S_{g}^{LDO}$ defined by:

$$S_{g}^{LDO}=S_{g}-S_{CN}(x_{g}|T^{LDO})$$

In Fig 1B, the essentiality score before and after LDO correction is shown for the *YAP1* amplicon in SF268. In Fig 1B (left), *ANGPTL5*, *KIAA1377*, *C11orf70*, *BIRC3*, *BIRC2*, *TMEM123*, *MMP7*, and *MMP20* display equivalently significant phenotypes believed to be entirely due to the copy number artefact. On the other hand, YAP1 shows a stronger phenotype relative to its neighbouring genes.

In Fig 1B (right), the resulting corrected sensitivity scores are shown in the *YAP1* amplicon in SF268. The copy number effect has been successfully removed and *YAP1* still scores as significantly lethal, thereby being identified as the amplicon’s driver, as is expected from existing shRNA screens and reported elsewhere [13,14].

Overall, LDO removes the copy number effect beyond the *YAP1* amplicon in SF268 and in MKN45 cell lines, as shown in Fig 1C. The number of guides with log2(CNA) larger than 2 and LogFC below -0.5 is decreased from 98 to 37 guides in MKN45 and 267 to 38 guides in SF268. Finally, and although this is not the main motivation for the development of this method, the LDO strategy can be used to predict copy number alterations in the screened samples (see S3 Fig).

### Library design and guide quality

Although the method applied in this screen was able to successfully recover the driver in the *YAP1* amplicon, this is not always the case as shown in Fig 2A.

**Fig 2:**
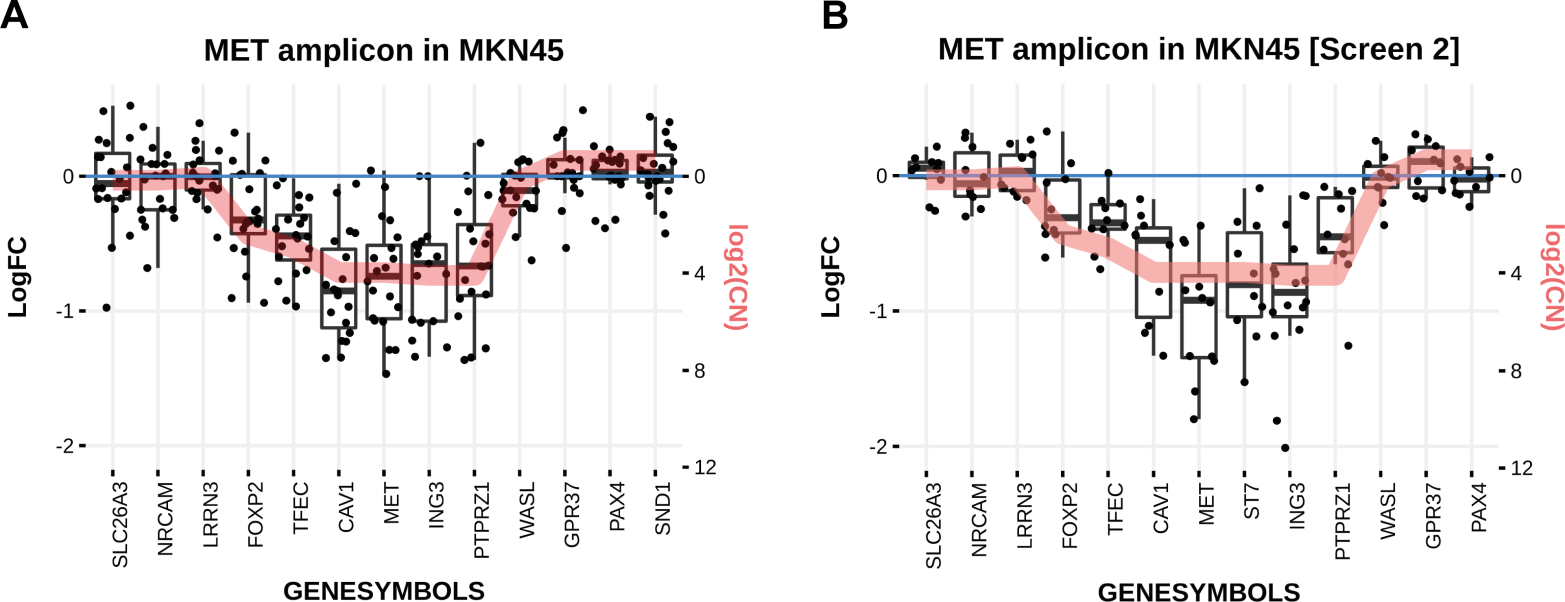
MET Specific LDO correction in MKN45 in two different screens. **A)** Sensitivity conferred by each guide (black dots) within the MET amplicon in MKN45 summarized by a boxplot for each gene in the amplicon. The red line displays the inverted copy number value scaled to the data. **B)** Sensitivity conferred by each guide (black dots) within the MET amplicon in MKN45 summarized by a boxplot for each gene in the amplicon. The red line displays the inverted copy number value scaled to the data.

From shRNA screens and other reports [15,16], *MET* is expected to be the driver of this amplicon. Therefore, one could expect the *MET* guides to display a stronger relative drop out compared to the rest of the genes in the amplicon. However this was not the case and thus applying the LDO correction did not enable the recovery of *MET* as the driver of the amplicon. The degree of amplification does not appear to explain the lack of differential *MET* effect in MKN45 considering that the amplification in SF268:*YAP1* is equivalent to what is seen in MKN45:*MET*.

One potential reason for this lack of relative drop out is the quality of the guides used. The screen was rerun with different guide designs. The result for the *MET* amplicon in sample MKN45 is shown in Fig 2B and in this case, *MET* does display a stronger phenotype than the rest of the amplicon. This highlights the need for careful library design (S1 Fig).

### Application of the LDO method on the Avana data set

To verify the generalizability of this method we applied LDO to the Avana data set of 342 cell lines screened with a genome-wide CRISPR library [11]. We used the guide level dependency scores as well as the CCLE [19] copy number provided by Meyers & al. The guide scores were further scaled so that the mean guide scores targeting nonessential and essential genes are equal to 0 and -1 respectively in each sample. The reference set of nonessential and essential genes established in [20] was used for the scaling.

In Fig 3A we show that in the Avana data set LDO again markedly decreases the correlation between gene dependency and copy number compared to the uncorrected data. To assess the risk of over correction by LDO, we considered the recall of the guides targeting essential, nonessential and CN amplified regions (i.e. regions with copy number > 5). A similar strategy was used in [12]. In Fig 3B the recall curves for cell line DAN-G is presented before and after LDO correction. We notice that the recall for the essential genes is barely affected by the correction, indicating that the LDO method does not markedly impact the sensitivity to detect these genes. The recall curve for the nonessential group of genes is also not strongly affected by the correction as opposed to the recall of the amplified genes which is strongly reduced in DAN-G. To assess these recall curves across the whole dataset the area under the recall curve (AURC) was used. In Fig 3C the AURC before and after LDO correction is presented for all samples. The AURC for the essential genes remains unaffected by the correction while the AURC for the nonessential genes is slightly increased. The median AURC across samples is shifted from 87.3 to 87.1 and from 41.8 to 44.6 for the essential and nonessential genes respectively. This is in contrast to the shifts in median AURC observed for the amplified genes from 62.5 to 50.5 when using all samples or from 77.5 to 52.6 when considering only samples with at least 25 genes in amplified regions.

**Fig 3:**
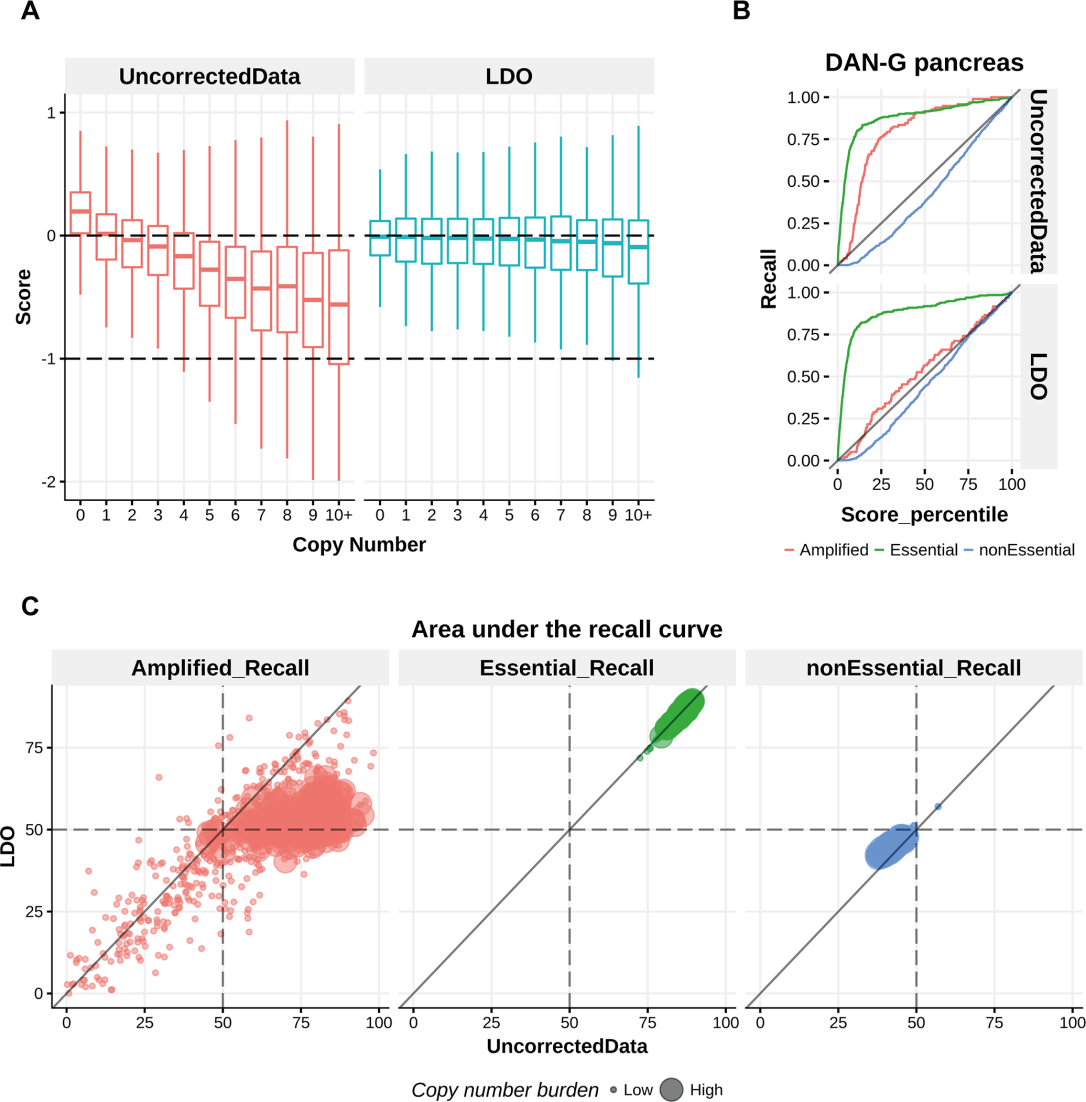
LDO removes the copy number effect across samples and maintains sensitivity of essential genes. **A)** Boxplot of dependency scores across copy number for uncorrected and LDO corrected data. **B)** The recall curve for essential, nonessential and amplified genes is shown before and after LDO copy number correction in the cell line DAN-G. **C)** The area under the recall curve is shown across samples for the essential, nonessential and amplified genes.

### Generalized Additive Model (GAM) method

In contrast to the LDO method, the GAM method is a supervised strategy requiring orthogonal data, such as copy number for the correction. To do this, we used a generalized additive model (GAM, [21]) framework and modelled the sensitivity to CRISPR-mediated gene knock-out as a function of copy number to yield an adjusted CRISPR-mediated gene knock-out estimate. In addition to its ability to leverage the copy number values when available, the potential benefit of this framework compared to LDO is that it can be extended to consider any arbitrary number of additional features (both linked to artefactual or true effects) potentially relevant for the purpose of modelling the phenotype (e.g. gene expression, multi-alignment of guides, etc.). In this analysis, only the copy number measurements were used. Once the model has been fitted, the effect of the artefactual components of the sensitivity can be removed from the observed phenotype in order to keep the “biologically-relevant” component (in this example only the artefactual copy-number effect is considered and removed). Unlike the LDO method, the GAM method is insensitive to the screen density and would be preferred should a sparse coverage of the genome be considered in the screen. Additionally the GAM method does not require a prior list of known essential genes to be performed.

The GAM structure can be written as follows:

$$E(S_{g})=\alpha +s_{1}(x_{1}^{g})+\cdots +s_{p}(x_{p}^{g})$$

Where *E*(*S*_*g*_) is the expected sensitivity of guide *g*; $x_{1}^{g},\ldots ,x_{p}^{g}$ are the predictor variables for *g* and $s_{1}(x_{1}^{g}),\ldots ,s_{p}(x_{p}^{g})$ denote the smoothing functions estimated by non-parametric means from the data. Finally, *α* is the intercept. Note the lack of a linker function in the above equation compared to the canonical GAM framework, since we consistently use the identity function. For the purpose of fitting the GAM to the data, we use the R implementation from the mgcv package [22] with default parameters, so that penalized thin plate regression spline models are used for the smoothing.

This framework enables us to take into account an arbitrary number of predictor variables to model both linear and non-linear dependencies of the data. The aim is to remove from the measured sensitivity *S*_*g*_ the components of the model, which are deemed to come from artefactual predictor variables (e.g. copy number) but keep those coming from variables which are considered true predictors of biological sensitivity (e.g. gene expression). Instead of $x_{1}^{g},\ldots ,x_{p}^{g}$ let us further differentiate the predictor variables into the artefactual variables ${\hat{x}}_{1}^{g},\ldots ,{\hat{x}}_{l}^{g}$ and the explanatory variables $x_{l+1}^{g},\ldots ,x_{p}^{g}$ so that the GAM corrected sensitivity can be written as

$$S_{g}^{GAM}=S_{g}-\sum_{i=1}^{l}s_{i}({\hat{x}}_{i}^{g})$$

In this presentation a single artefactual predictor ${\hat{x}}_{1}$ is used which represents the copy number value at the position of guide *g*. The GAM corrected sensitivity score $S_{g}^{GAM}$ can then be used in lieu of the original sensitivity score with the same hit-defining thresholds and interpretation.

The correction of the copy number artefact in SF268 and MKN45 using GAM is shown in S2 Fig.

## Discussion

The use of high-throughput CRISPR screens to identify cell autonomous cancer dependencies has become routine. However, as shown in previous studies, these screens display high rates of false positive hits in regions of high copy number amplifications. In this report, we describe two methods, Local Drop Out (LDO) and General Additive Model (GAM), to correct for this copy number bias, thereby enabling the identification of true positive hits while reducing false positives substantially. In both cases the methods were developed with experimental setups in mind utilizing only a few number of cell lines, including single model experiments. Thus, making these methods appropriate for a broad range of experiments. As a result the CN artefact corrections proposed are performed at the level of single samples. We applied both methods to previously published screening data of 2722 genes performed in the SF268 and MKN45 cell lines. The utility of the methods were shown by way of two examples: first, the *YAP1* dependency in SF268 was recovered, while removing 8 false positive genes from the hit list (*ANGPTL5*, *KIAA1377*, *C11orf70*, *BIRC3*, *BIRC2*, *TMEM123*, *MMP7*, and *MMP20*); second, the *MET* dependency in MKN45 was recovered in one of the two screens, while removing 3 false positive hits (*CAV1*, *ST7*, and *ING3*). Overall, the number of guides with log2(CNA) larger than 2 and LogFC below -0.5 is decreased from 98 to 37 guides in MKN45 and 267 to 38 guides in SF268 when using LDO; with GAM the number of guides are reduced to 28 and 37 guides for MKN45 and SF268 respectively.

Additionally the LDO method was applied to an external data set of 342 viability screens. There the method again markedly lowered the copy number effect on cell viability while retaining the sensitivity to known essential genes thus demonstrating the generalizability of the method to a larger data set.

These methods, however, do have limitations. We observed that rescuing true positives within amplicons is only possible if the driver mutation in the amplicon of interest is displaying a stronger drop out relative to the neighbouring genes. Depending on the guides used, this is not always the case as demonstrated with *MET* in MKN45 in our first screen. Despite this caveat, both methods are still able to remove false-positives, although the true positive is not rescued in this case. We would argue that in a typical screening effort, the loss of a few true positives is preferred to a large amount of false positives, as would be the case if one does not correct for the copy number effect. Indeed, a lot of effort and resources can be spent chasing an elusive false positive. The second obvious alternative is to remove any amplified region from the subsequent analysis, which means a large amount of false negative hits, since those would not even be considered for further analysis, but also relies on prior available copy number measurements which is not always the case.

Another limitation is that these methods are highly dependent on guide scores obtained in the screen which can be variable. In our MKN45: *MET* example, it is unclear what the reason for the difference in drop out of the driver is. Guide design could be an explanation, however if CRISPR genome editing does indeed generate two cellular responses in cancer cells as suggested in [6]: an early anti-proliferative DNA damage response and a later gene dependant effect, the number of doublings before harvesting could also be an explanation. Whereby cell lines with long doubling times would only undergo enough doublings to sustain the DNA damage response but not enough to signal a differential effect from the driver genes. This hypothesis however does not seem to fit with the doubling times of 29h and 44h for MKN45 and SF268 respectively as reported in the Cancer Cell Line Encyclopedia (CCLE) [19].

Outside of these limitations, each of the two methods presented offer different advantages to the correction for the copy number induced false positives in loss-of-function CRISPR screens. The LDO method can correct for the copy number artefact even when copy number is not known beforehand as long as the density of the CRISPR screen is high enough to capture the copy number events with confidence. As opposed to LDO which is unsupervised, the GAM correction is a supervised method requiring copy number measurements. It is however not dependent on high density screens and can additionally incorporate an arbitrary number of predictor variables in its model. As a supervised method GAM remains the appropriate method should the copy number information be available. The fact that LDO does not need any copy number information also enables the user to infer copy number alterations based on CRISPR screens by exploring the magnitude of the correction that was applied to the different genomic regions (S3 Fig).

## Methods

### Essential genes

To collect an initial list of essential genes the results from [23] was used. In particular the essentiality of each gene was established in [23] using a genome-wide single guide CRISPR screen in 4 cancer cell lines. The strength of the essentiality is reported as an adjusted p-value in the accompanying data. Here, the genes with a maximum adjusted p-value of 0.05 across all 4 cell lines are used as de facto essential genes if and only if the accompanying CRISPR score is also smaller than -1. This results in a list of 814 potential essential genes (S1 Table).

### LDO

The choice of the exponential decay function in the weighted mean calculation is arbitrary (and any weighing function can easily be used instead in the provided scripts). Any monotonously decreasing function could be used, or, for example, a simple sliding window. The size of the window, or the value picked for *ω* in the exponential decay case, should be chosen so as to borrow the information from as many genes as possible while still remaining within the bounds of the expected copy number event sizes that are expected to be observed. The exponential decay function has the advantage of putting more weight to the genes in the direct neighbourhood of the gene of interest and thus even if the size of the window considered is relatively large the estimate remains relatively robust.

Similarly the values for *α*_1_, *β*_1_, the minimum number of three genes per short CN event and the choice of only considering events with correction values larger than the 1.5 times the median average deviation of the background noise were chosen arbitrarily based on a priori expectation of the effects we wish to correct for.

## Supporting information

S1 Fig Library design. Sensitivity conferred by each MET targeting guides (dots) in MKN45 along the MET gene in the first vs the second screen.
(TIF)

S2 Fig Global GAM correction. The sensitivity to CRISPR-mediated knock-out after GAM correction is not dependent on the level of DNA amplification of the underlying genomic region anymore.
(TIFF)

S3 Fig Using LDO for CN inference. The observed underlying CN for each guide against the sensitivity score correction inferred by the LDO correction. The sgRNAs in red represent guides targeting pan lethal clusters while those in orange are targeting focal amplifications with less than three genes.
(TIFF)

S1 Table Essential genes. List of 814 essential genes.
(CSV)
