# Supplementary figures and images for: Correction of copy number induced false positives in CRISPR screens
Source: PLoS Comput Biol. 2018 Jul 19;14(7):e1006279. doi: 10.1371/journal.pcbi.1006279 (PMC6067744; doi:10.1371/journal.pcbi.1006279)

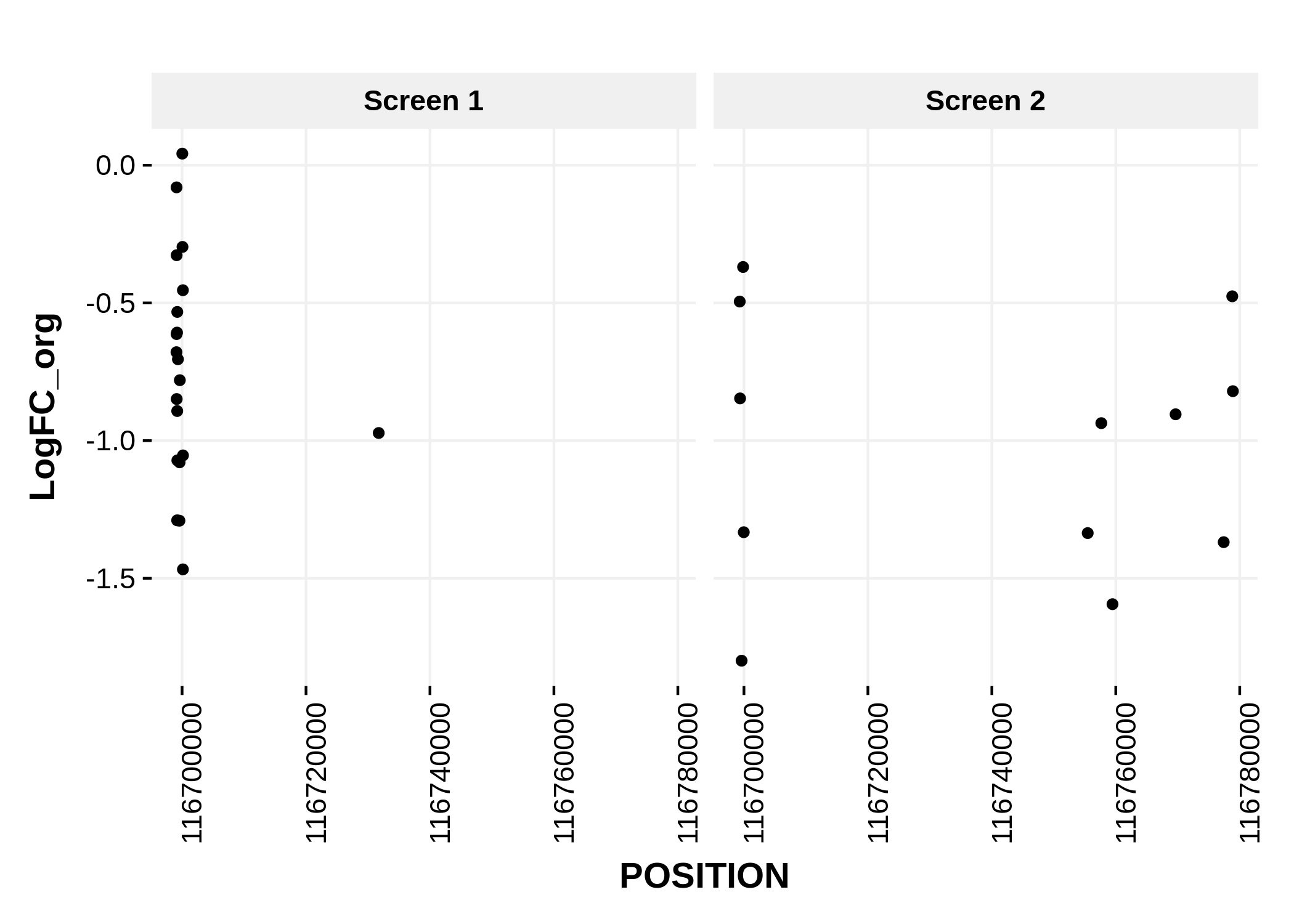

Supplement: S1 Fig — Sensitivity conferred by each MET targeting guides (dots) in MKN45 along the MET gene in the first vs the second screen. (TIF) [file pcbi.1006279.s001.tif]

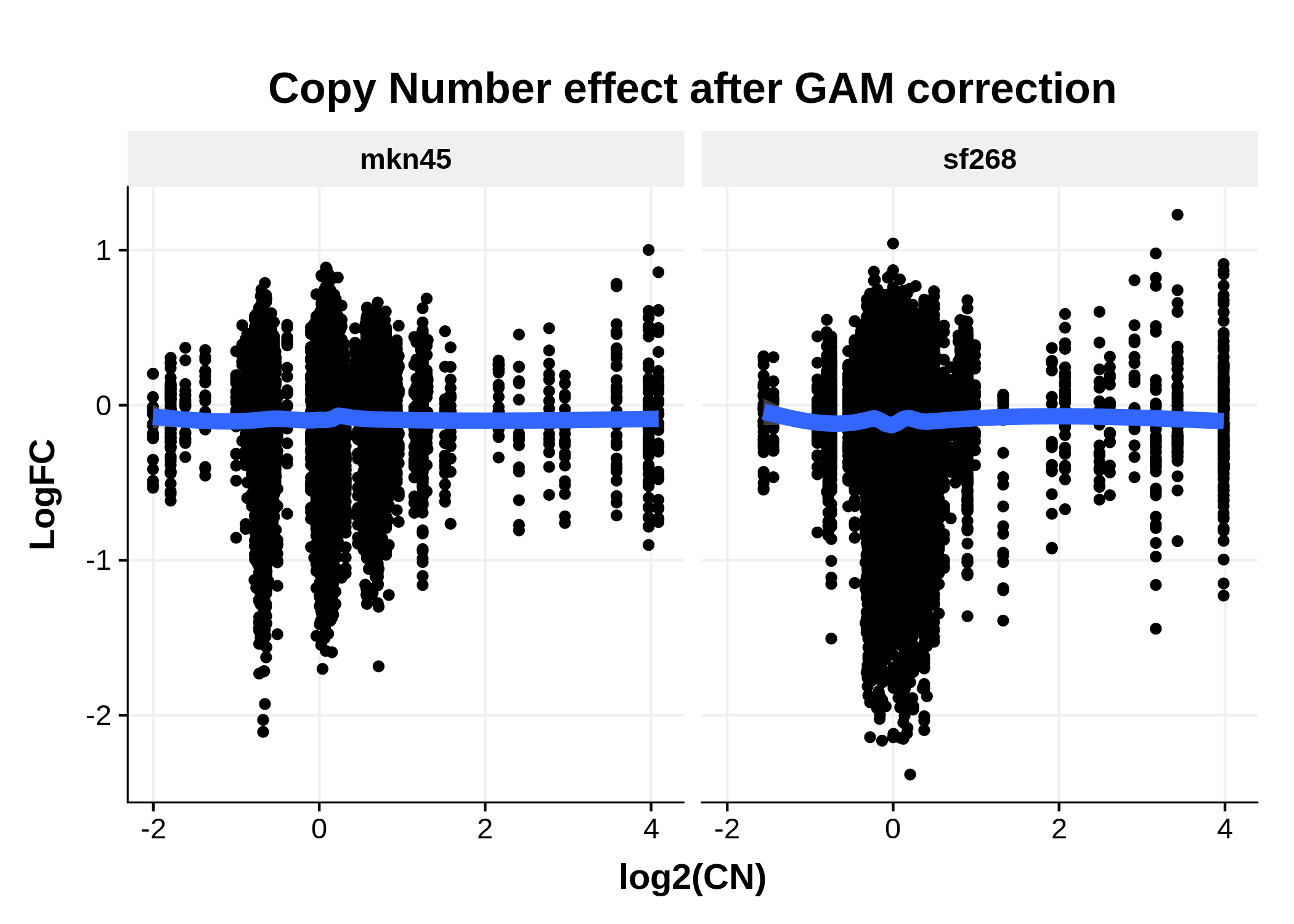

Supplement: S2 Fig — The sensitivity to CRISPR-mediated knock-out after GAM correction is not dependent on the level of DNA amplification of the underlying genomic region anymore. (TIFF) [file pcbi.1006279.s002.tiff]

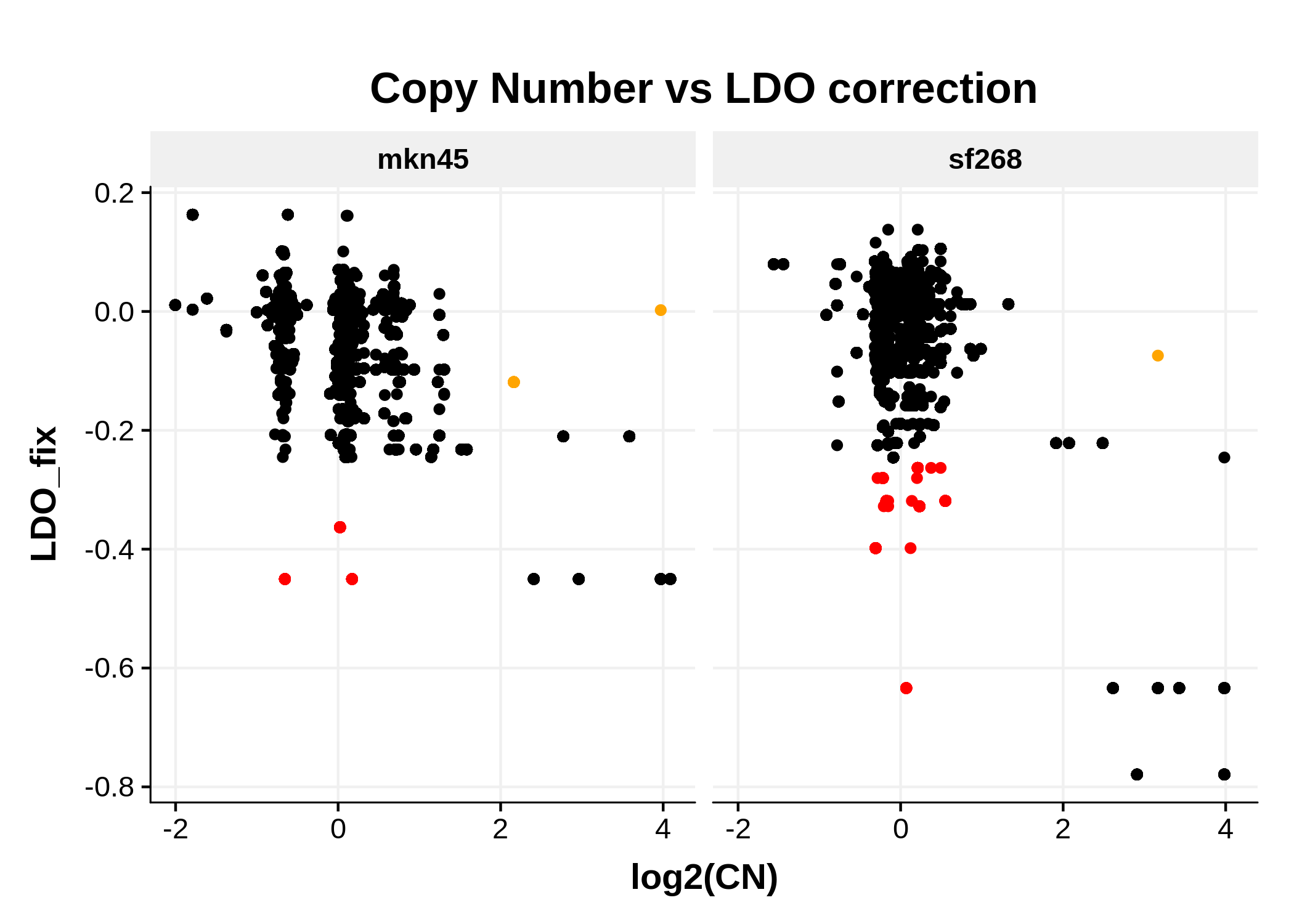

Supplement: S3 Fig — The observed underlying CN for each guide against the sensitivity score correction inferred by the LDO correction. The sgRNAs in red represent guides targeting pan lethal clusters while those in orange are targeting focal amplifications with less than three genes. (TIFF) [file pcbi.1006279.s003.tiff]
